# Screening in Gastrointestinal Malignancies—Recent Trials and Advancements

**DOI:** 10.3390/cancers17060975

**Published:** 2025-03-13

**Authors:** Natalia Czerw, Andrzej Deptała, Anna Badowska-Kozakiewicz, Aleksandra Czerw, Olga Partyka, Monika Pajewska, Katarzyna Sygit, Magdalena Michalska, Izabela Gąska, Grażyna Dykowska, Zofia Sienkiewicz, Elżbieta Grochans, Szymon Grochans, Anna Cybulska, Daria Schneider-Matyka, Ewa Bandurska, Weronika Ciećko, Jaroslaw Drobnik, Piotr Pobrotyn, Joanna Furtak-Pobrotyn, Dagmara Pokorna-Kałwak, Urszula Grata-Borkowska, Michal Marczak, Petre Iltchev, Remigiusz Kozlowski

**Affiliations:** 1 Students’ Scientific Organization of Cancer Cell Biology, Department of Oncology Propaedeutics, Medical University of Warsaw, 01-445 Warsaw, Poland; 2 Department of Oncology Propaedeutics, Medical University of Warsaw, 01-445 Warsaw, Poland; 3 Department of Health Economics and Medical Law, Medical University of Warsaw, 01-445 Warsaw, Poland; 4 Department of Economic and System Analyses, National Institute of Public Health NIH-National Research Institute, 00-791 Warsaw, Poland; 5 Faculty of Medicine and Health Sciences, Calisia University, 62-800 Kalisz, Poland; 6 Medical Institute, Jan Grodek State University in Sanok, 38-500 Sanok, Poland; 7 Institute of Nursing, College of Engineering and Health, 02-366 Warsaw, Poland; 8 Department of Nursing, Social and Medical Development, Medical University of Warsaw, 01-445 Warsaw, Poland; 9 Department of Nursing, Faculty of Health Sciences, Pomeranian Medical University in Szczecin, 71-210 Szczecin, Poland; 10 Department of Pediatric and Oncological Surgery, Urology and Hand Surgery, Faculty of Medicine and Dentistry, Pomeranian Medical University in Szczecin, 71-252 Szczecin, Poland; 11 Center for Competence Development, Integrated Care and e-Health, Medical University of Gdansk, 80-204 Gdansk, Poland; 12 Department of Family Medicine, Faculty of Medicine, Wroclaw Medical University, 51-141 Wroclaw, Poland; 13 Pulsantis Specialist and Rehabilitation Clinic Ltd., 53-238 Wroclaw, Poland; 14 Citodent Dental Center Furtak-Pobrotyn & Company Limited Partnership, 05-220 Olawa, Poland; 15 Department of Innovation, Merito University in Poznan, 61-895 Poznan, Poland; 16 Department of Management and Logistics in Healthcare, Medical University of Lodz, 90-131 Lodz, Poland

**Keywords:** gastrointestinal cancer, GI, screening, early detection

## Abstract

Gastrointestinal cancers, especially colorectal cancer, represent a major health care issue. According to the data, the incidence of these cancers exceeds 50/100,000, mainly in high-income countries. Risk factors for the disease include genetic factors (i.e., Lynch syndrome) and lifestyle factors, e.g., overweight and obesity, smoking tobacco products, low-fiber diet, and low physical activity. When diagnosed early, gastrointestinal cancers can, in most cases, be effectively treated. This review focuses on the presentation of research in the area of screening and its results. It provides insights into current trends in research on methods of early cancer detection and improving screening reporting. Differing levels of screening reporting are one of the main challenges faced by researchers in this field. Research on this issue should be conducted in parallel with research on methods of early diagnosis. Finally, the development of diagnostic methods and communication with patients are important elements of public health.

## Simple Summary

The incidence of gastrointestinal cancers in many high-income countries exceeds 50/100,000. Although patient health awareness in these countries is high and access to health care is universal, there are still significant gaps in screening reporting. Improving these rates is particularly important for cancer, one of the leading causes of death today, most of which can be effectively treated if detected early enough. The present review focuses on presenting trends among studies in this area.

## 1. Introduction

The World Health Organisation’s 2022 global cancer statistics report documented 511,054 cases of esophageal cancer (365,225 in the group of males and 145,829 in the group of females), 968,784 cases of stomach cancer (627,458 in the group of males and 341,326 in the group of females), 1,142,286 cases of colon cancer (609,228 in the group of males and 533,058 in the group of females), 1,926,425 cases of colorectal cancer (1,069,446 in the group of males and 856,979 in the group of females), 729,833 cases of rectal cancer (436,185 in the group of males and 293,648 in the group of females), and 54,306 cases of anal cancer (24,033 in the group of males and 30,273 in the group of females) [1].

Age-standardized incidence rates were as follows: esophageal cancer—5.0/100,000 (7.6/100,000 in the group of males and 2.6/100,000 in the group of females); gastric cancer—9.2/100,000 (12.8/100,000 in the group of males and 6.0/100,000 in the group of females); colon cancer—10.7/100,000 (12.4/100,000 in the group of males and 9.2/100,000 in the group of females); colorectal cancer—18.4/100,000 (21.9/100,000 in the group of males and 15.2/100,000 in the group of females); rectal cancer—7.1/100,000 (9.1/100,000 in the group of males and 5.4/100,000 in the group of females); and anal cancer—0.54/100,000 (0.51/100,000 in the group of males and 0.57/100,000 in the group of females). Gastrointestinal cancer incidence rates surpass 50/100,000 in Australia, France, Hungary, Ireland, Mongolia, the Netherlands, Norway, Portugal, Romania, and Slovakia.

Global age-standardized mortality rates are 4.3/100,000 for esophageal cancer (6.5/100,000 in the group of males and 2.2/100,000 in the group of females), 6.1/100,000 for stomach cancer (8.6/100,000 in the group of males and 3.9/100,000 in the group of females), 4.7/100,000 for colon cancer (5.6/100,000 in the group of males and 4.0/100,000 in the group of females), 8.1/100,000 for colorectal cancer (9.9/100,000 in the group of males and 6.5/100,000 in the group of females), 3.1/100,000 for rectal cancer (4.1/100,000 in the group of males and 2.3/100,000 in the group of females), and 0.21/100,000 for anal cancer (0.22/100,000 in the group of males and 0.20/100,000 in the group of females).

According to data from the European Commission [2], the five-year survival probability was highest for colon cancer in the 15–44 age group (63.36%), in the 65–74 age group (56.76%), and in the 75+ age group (49.23%), and for rectum cancers in the 45–54 (61.24%) and 55–64 (60.69%) age groups. The five-year survival probability was lowest for esophagus cancer, ranging between 19.48% in the 15–44 age group and 6.12% in the 75+ age group. The five-year survival probability for various cancers is generally higher in females than in males across all age groups. The biggest difference is in esophageal cancer in the 15–44 age group. The age-specific five-year relative survival for males is equal to 17.77% and to 26.62% for females. However, there are exceptions. For colon and rectum cancers, the age-specific five-year relative survival for males is equal to 47.35%; it is slightly higher than for females, for whom it is equal to 47.28%. For esophageal cancer, the five-year survival rate is higher in males than in females in the oldest age group (75+), with rates of 6.28% for males compared to 5.88% for females. Similarly, for rectal cancer in the same age group, the survival rate for males is also higher, at 44.86%, compared to 43.90% for females.

Risk factors for colorectal cancer, the most common type of gastrointestinal cancer, include the following: being overweight or obese, particularly in men; having type 2 diabetes; consuming high amounts of red and processed meats; eating fried and grilled foods; having low levels of vitamin D; and engaging in smoking and excessive alcohol consumption. Additional risk factors include being over the age of 50, having a history of cholecystectomy (gallbladder removal), a personal history of adenomatous polyps, and a family history of colorectal cancer or adenomatous polyps. Furthermore, mutations in the hMLH1 and hMSH2 genes, commonly associated with Lynch syndrome [3], also increase risk. According to an umbrella review of various studies, a diet rich in fiber, dairy, fruits, and vegetables can help reduce the risk of gastrointestinal cancers [4].

Diagnoses and screenings for gastrointestinal cancer utilize endoscopic examinations, biopsies, and imaging modalities such as ultrasound, computer tomography (CT), magnetic resonance imaging (MRI), and positron emission tomography (PET).

The U.S. Preventive Services Task Force (USPSTF) guidelines advise colorectal cancer screening for the 45–49 and 50–75 age groups; for individuals aged 76–85, it is advised to offer screening for colorectal cancer only selectively due to the small net benefit of screening in this age group [5]. Examinations of patients’ overall health, as well as prior screenings conducted and their differences, should be taken into account when deciding in this regard. USPSTF summarizes evidence that the mortality rate is lower in people who have undergone at least one screening colonoscopy compared with those who have never had a colonoscopy (adjusted hazard ratio, 0.32 (95% CI, 0.24–0.45)) [6]. Conducting flexible sigmoidoscopy was also associated with a decrease in mortality (IRR, 0.74 (95% CI, 0.68–0.80), 10 to 17 fewer CRC deaths/100,000 person-years) when compared with no screening at of 11 to 17 years of follow-up [7]. Finally, screening with a fecal immunochemical test was associated with lower mortality at 6 years’ follow-up when compared with the no screening group (adjusted RR, 0.90 [95% CI, 0.84–0.95] [8].

## 2. Materials and Methods

This paper aims to present current trends in clinical trials for screening gastrointestinal malignancies.

Our analysis is based on information from the world’s largest clinical trials registry (https://clinicaltrials.gov/). This registry is maintained by the U.S. National Institutes of Health and the National Library of Medicine. ClinicalTrials.Gov is the most extensive database, featuring over 507,000 clinical trials conducted in over 220 countries [9]. Due to the topic covered, only studies addressing cancers of the gastrointestinal tract, such as esophageal cancer, gastric cancer, small bowel cancer, colorectal cancer, colon cancer, rectal cancer, and anal cancer, were included in the analysis.

Both interventional and observational studies related to gastrointestinal cancer screening were considered. The search period was limited to 2019–2024 to reflect current trends in research. Only complete studies with defined outcomes and active statuses on 20th November 2024 were included. Also, we limited the studies included to the those that had the results posted for examination.

**Figure 1:**
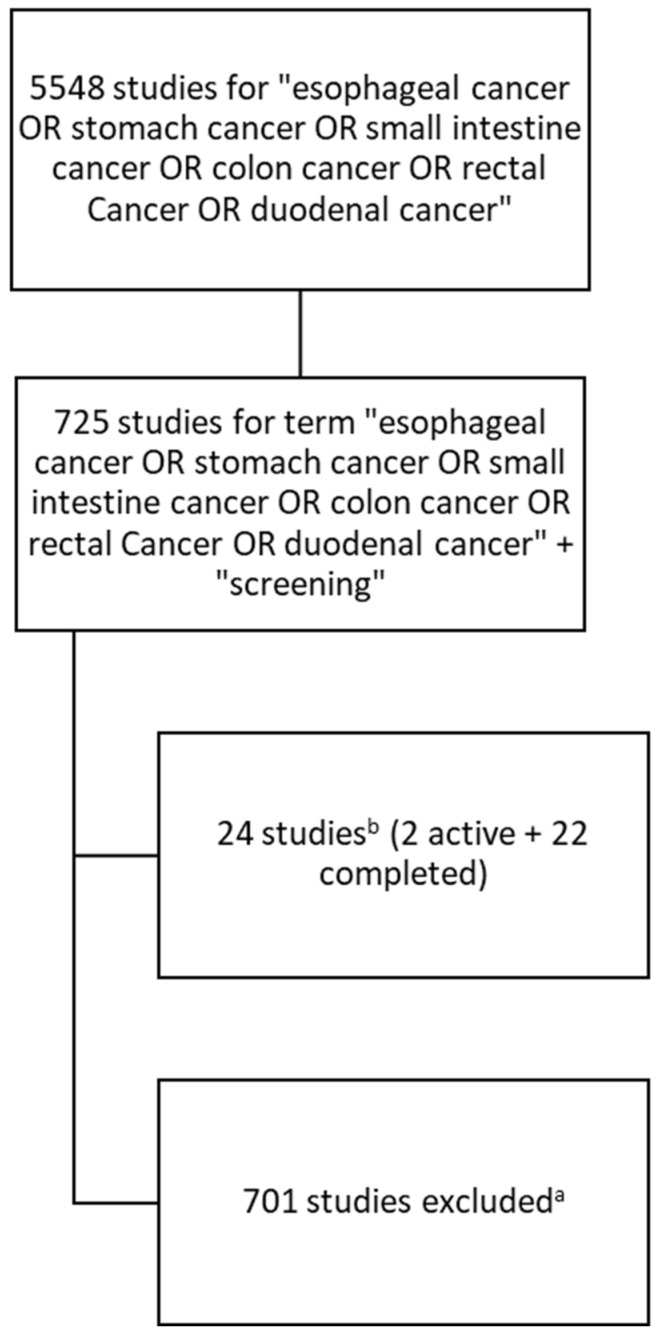
Scheme for identifying and incorporating studies into the analysis. ^a^—studies with status: without results; ^b^—with results in the 1 January 2019–20 November 2024 period.

The procedure for incorporating studies into the analysis is presented below (Figure 1):

We started with a query listing of the types of cancers that were our focus as a condition/disease filter; then, we added “screening” as an additional keyword, and finally, we limited the list of the studies registered to those that had posted results, thereby excluding studies without results.

## 3. Results

A data search identified 24 studies; however, five focused on treatment methods for patients diagnosed with gastrointestinal cancer. These studies were obtained in response to a query, as testing the effectiveness of new treatments requires systematic monitoring of patients’ conditions, also referred to as “screening”. These studies were excluded from the presented review. Of the remaining 19 studies, one was active but with the results posted, while 18 had been concluded.

Table 1 presents the analysis results of digestive system cancer screening studies conducted during the specified study period.

**Figure 2:**
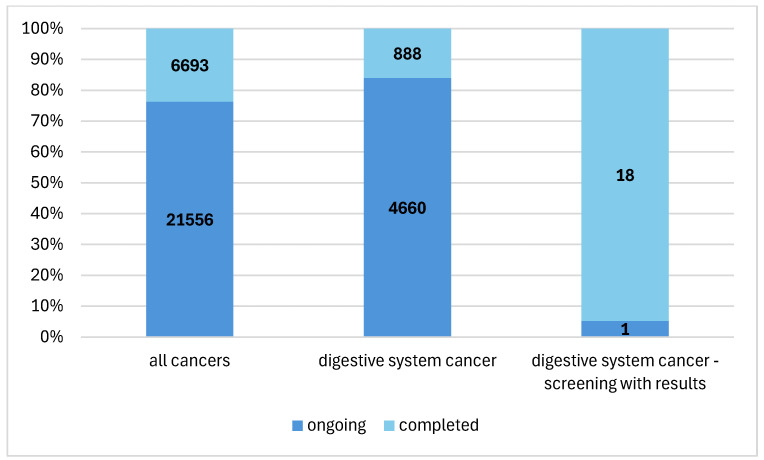
The percentage of cancer of the digestive system screening studies in the context of overall cancer research.

From 28,249 cancer studies registered between 2019 and 2024, gastrointestinal cancer studies represented 19.6% (*n* = 5548) of the overall database (Figure 2). Among these, digestive system cancer screening studies comprised 13.1% (*n* = 725) of all digestive system cancer studies, but the results regarding screening were posted from only 19 studies (3.3%).

Of the nineteen studies conducted, two commenced in 2019, nine in 2020, six in 2021, and two in 2022. One study was observational, while the other eighteen were intervention studies. Eighteen of the studies focused exclusively on adults. The sample sizes varied significantly, ranging from 6 to 77,145 participants. Fourteen studies were behavioral interventions, and the remaining five involved diagnostic procedures. Eighteen studies included both females and males. One study focused on females exclusively.

### 3.1. Behavioral Interventions

A study from 2020–2022 (1) demonstrated a greater than 2.4-fold increase in the likelihood of colorectal cancer screening participation among those also screened for social determinants of health. The conditional odds ratio for participating in colorectal cancer screening was equal to 0.068 in the group with social determinants of health assessed and to 0.028 in the group undergoing a standard procedure only. The study involved 26 community health center staff members who had access a large patient population of 60,607. The colorectal cancer screening method examined in this study was stool immunochemical analysis [10].

The provision of self-administered stool immunochemical test kits for colorectal cancer screening proved to be considerably more effective in encouraging patient participation than disseminating information promoting screening (2). The effect of providing self-administered test kits was very large. As much as 75% of patients used the kit they received, which was compared to only 1% of patients who followed the usual recommendations and participated in testing. The study was performed in rural areas [11].

The effectiveness of sending out kits for self-administered fecal immunochemical testing for colorectal cancer was also demonstrated in a study involving men and women in two age groups, i.e., 45–49 years and 50–75 years (3). The findings also indicated that more patients who received a test kit underwent a colonoscopy. The differences were smaller than in the study mentioned above, but still significant. Specifically, the effect of the intervention was stronger in the 50–75 age group (29.9% vs. 9.6% of patients who participated in the diagnostics following the intervention) and weaker in the 45–49 age group (33.5% vs. 28.5% of patients who participated in the diagnostic procedure following the intervention) [12].

Another study (4) analyzed the percentage of patients who performed immunochemical stool testing based on the method of invitation to participate in the study. It revealed that, among individuals aged 50–74 years, reminders to undertake the test sent via email or letter were the most effective, i.e., 21.2% and 20.3% of patients participated in the screening after reminders were sent electronically or by letter respectively. In contrast, simply sending a self-test kit without reminders to use it was less effective, i.e., 14.6–18.0% of patients participated in the screening and used the kit provided [13].

A subsequent study (5) examined the use of an app for collecting diagnostic data concerning colorectal cancer diagnosis before medical appointments. An analysis was conducted to determine whether higher or lower levels of interaction with patients and medical staff yielded greater success in encouraging app utilization. The study population comprised healthcare professionals and patients aged 18 years and 50–74 years who presented with indications for colorectal cancer diagnosis. The published results show no significant differences. Only 0.9% of patients who completed a higher intensity program vs. 1.0% of patients who completed a lower intensity program used the application [14].

A higher percentage of patients agreed to participate in a colonoscopy when they were involved in shared decision-making processes that clearly outlined the study’s benefits and potential negative consequences (6). The research involved patients who could not participate because of COVID-19 restrictions. The effect observed was significant. In the group patients participating in the shared decision-making process, 35.1% underwent a colonoscopy, while in the control group, the percentage of patients having a colonoscopy was equal to 22.8% [15].

A 2021–2023 study (7) verified the efficacy of personalized colorectal cancer screening messages sent to seniors. Personalized messages that assess the risks and consequences of the disease, along with explanations of the benefits and potential side effects of participating in a colonoscopy and stool immunochemical tests, were shown to increase the percentage of participation in diagnostic tests. Additionally, the extent to which caregivers were informed about the option to participate in these tests was also an essential factor. However, the effects were rather weak: 35.1% of individuals decided to participate in screening in the group of patients that received personalized screening messages, and 31.0% of individuals decided to participate in screening in the group of patients that only received information material [16].

In the next study, a comparison of patient involvement in screening decision-making was conducted across three groups: breast, colorectal, and prostate cancer screening participants (8). In this study a decision participation scale was used; it measured the patient involvement on an interval scale. Therefore, the calculation of arithmetic means was possible. The degree of patient involvement in decision-making in the group diagnosed with colorectal cancer was lower than in the group diagnosed with prostate cancer. The value of Cohen’s d effect size measure was equal to d = 90; this value is interpreted as indicating for a large effect [17,18].

A study of 115 African-American adults (9) found that the involvement of community health counsellors surprisingly did not increase the percentage of patients who underwent a colonoscopy or immunochemical stool test compared to situations where the immunochemical stool test kit and information materials were mailed to participants. The majority, i.e., 64.4%, of patients in the group who met with the counsellor completed the study. However, the rate, equal to 64.6%, was as high or even slightly higher in the control group [19].

A study involving 42 Hispanic adults (10) found that an interactive app encouraging colonoscopy registration was surprisingly not more effective than a video providing general health information. The results showed that 86.4% of patients presented for colonoscopy in the interactive app intervention group. The effect of providing information on a video was actually better, because 95.0% of patients in the control group participated in the diagnostic colonoscopy after watching the video [20].

The effectiveness of videos was also demonstrated in a study in which residents of remote high-altitude towns were given immunochemical stool testing kits and text, audio or video instructions (11): 15.0% of patients took the test when provided with a text instruction, 9.5% of patients took the test when provided with an audio instruction, and 28.1% of patients took the test when provided with a video instruction [21].

The next study examined whether the level of interactivity of online information (ranging from passive websites to virtual assistants) affected patients’ perceptions of their colorectal cancer risk, their intention to participate in diagnostic tests, and their interest in seeking further information about cancer diagnosis (12). However, no significant differences were identified. The intention to take part in diagnostic tests was measured on an interval scale which made calculating arithmetic means for the groups of patients possible. The value of Cohen’s d effect size measure for the difference between the group using the website and the group using the virtual assistant was equal to d = 0.13, which is interpreted as a minimal effect strength [22].

In another aspect of the study, interventions that involved delivering informational materials to patients’ homes and assisting in scheduling medical appointments for colonoscopies or fecal examinations were deemed helpful by more than 84% of patients out of 183 patients aged 50 to 75 years (13) [23].

Additionally, a separate study explored the rates of HPV testing and anoscopy participation among men who have anal sex with men (14). It was found that a higher percentage of men who received an HPV self-testing kit opted for testing compared to those who were offered the test in a medical facility. The difference was between a 65.0% rate of performing the test in the group of men who received a kit to be used at home and a 52.5% rate of performing the test in a medical facility. Interestingly, among those who had the option to test at a medical facility, a significant number participated in an anoscopy; this number was higher than in the group of men who received the test to be used at home, i.e., 63.4% and 51.2% respectively [24].

The primary outcome measures applied in the majority if the studies verifying behavioral interventions were based on proportions of patients who participated and completed a diagnostic examination. Also, scores acquired with the use of questionnaires, like Shared Decision Making (SDM) Process Scale Score and 7-point Likert scales, were used.

### 3.2. Diagnosing

One study also addressing the cohort of men who have anal sex with men (15) found at least a 95% concordance between patient-administered palpation tests following training and those performed by medical professionals. Furthermore, there was a minimum of 88.5% concordance between palpation tests performed by patients’ partners, post-training, and those conducted by medical professionals. It is also worth noting that this interventional study was based on a sample of 718 males who had anal sex with males aged 25 years or more. The large sample supports the credibility of the results acquired [25].

Additionally, a study on the diagnostic management of gastroesophageal reflux disease and Barrett’s esophagus (16) indicated that transnasal endoscopy led to significantly more patient-reported pain and a decreased willingness to undergo repeat procedures in the future compared to the use of cytosponge and traditional gastroscopy. The pain was assessed with the use of the pain analogue scale (VAS), which is an interval scale. The value of Cohen’s d effect size measure was equal to d = 1.51 for the difference between the group in which transnasal endoscopy was performed and the group in which cytosponge was utilized and to d = 2.01 for the difference between the group in which transnasal endoscopy was performed and the group in which traditional gastroscopy was performed. Both effects were very large. Regarding the willingness to undergo repeat procedures in the future, the percentage of patients willing to repeat the examination in the future was equal to 87.5% in the group with transnasal endoscopy and to 100% in the other two groups [26].

An analysis was conducted to evaluate the efficacy of oncologist training programs regarding patient referrals for genetic testing of colorectal, pancreatic, and other cancers (17). Post-training, oncologists referred 25 out of 48 qualifying patients, compared to 29 out of 100 pre-training, which gives rates of correct medical decisions of 52.1% and 29.0% respectively. However, the study was conducted on a small group of six oncologists only [27].

The next study evaluated the application of optical coherence tomography (OCT) during colonoscopy (18) to improve the accuracy of visible lesion depth assessment, informing choices between endoscopic or surgical excision of changes. The concordance between OCT-guided colonoscopy and biopsy results measured by κ Cohen’s coefficient was high, i.e., κ = 0.85. The high value of κ Cohen’s coefficient indicates high reliability and validity of the method under evaluation. However, this single study was still ongoing with, a sample size equal to 36 cases, when this review was prepared, so additional results are expected in the near future [28].

A high sensitivity (19) for colorectal cancer detection was demonstrated using fecal RNA testing. The sensitivity of the test validated against colonoscopy results was 92.6%. This interventional study was based on a very large sample of 14,263 cases of adults aged 45 or more. The large sample size is a basis for high credibility of the provided results [29].

Primary outcome measures applied in the studies verifying diagnostic methods were based on concordance and sensitivity measures, but also on the scores acquired with the use of questionnaires like the pain analogue scale.

## 4. Discussion

A significant majority of the reviewed studies (16 out of 19) focused on colorectal cancer, which is the most common gastrointestinal malignancy. Colonoscopy is the most widely used diagnostic procedure for this type of cancer; however, seven out of fifteen studies on colorectal cancer diagnosis employed immunochemical stool testing.

The findings from studies on the effectiveness of behavioral interventions suggest that when patients are involved in decision-making regarding their diagnostic management, their motivation to participate in research increases. Interestingly, sophisticated interactive information technology is not necessary to achieve this motivation, as indicated by the results of three different studies. This may be an interesting conclusion from an economical point of view, as applying expensive new technologies is not necessarily effective regarding behavioral interventions to enhance patients motivation for participating in diagnostics.

Most studies focused on behavioral interventions aimed at increasing patients’ motivation to participate in diagnostic testing. However, two studies specifically evaluated new diagnostic methods: optical coherence tomography (OCT), used during colonoscopies, and an RNA test for stool examination. Both studies were related to the diagnosis of colorectal cancer. The results provided proved each of the two methods to be reliable; the study verifying the use of OCT was still ongoing though, and the sample size was rather small.

Certainly, our review has limitations. Firstly, it was limited to the studies registered on clinicaltrials.gov only. All but one of the studies included in our review were located in United States; one study was located in China. The locations did not cover many geographical regions and did not correspond to the prevalence rates of gastrointestinal malignancies in those regions. Also, the years between 2019 and 2024 were affected by the COVID-19 pandemic, during which patients’ willingness to participate in research studies may have been limited.

Notably, there were no studies on gastric cancer despite its inclusion in the search criteria for published results from 2019 to 2024. Gastric cancer has a high mortality rate, second only to colorectal cancer among those analyzed, yet it seems to draw little interest from researchers who registered their studies on clinicaltrials.gov. For the latest review regarding screening methods for gastric cancer, the reader should refer to a systematic review and meta-analysis focused on the efficacy, effectiveness, and cost-effectiveness of endoscopic screening in intermediate risk countries [30]. Also, a review and meta-analysis evaluating the diagnostic value of cfDNA-based markers for gastric cancer was recently published [31]. The hope is to develop a method to detect circulating cell-free DNA to provide a more economical and less invasive alternative to gastroscopic screening. The pooled specifity acquired in the meta-analysis was very high. The sensitivity for the currently available test was less satisfying. However, cfDNA may be considered as a possible replacement for endoscopic screening in the future.

In Poland, the National Cancer Strategy includes a screening program aimed at the early detection of colorectal cancer, the most common cancer of the digestive system. As part of this program, preventive colonoscopies are conducted. However, no specific behavioral interventions or novel imagining diagnostic tools are involved. Eligible participants are individuals aged 50 to 65 years or those aged 40 to 49 years who have a first-degree relative diagnosed with colorectal cancer, so the inclusion criteria are, to some extend, different to the ones provided in USPSTF guidelines. The Polish Screening Programme is focused on the 50 to 65 years age group. Younger patients are entitled to participate provided that colorectal cancer has been diagnosed in an immediate family member.

**Table 1:** Distribution of clinical trial analysis results in the clinicaltrials.gov registry by adopted categories.

No.	Group	Title	*n*	Status	Study	Population	Effect Strength
1.	Interventions	Paired Promotion of Colorectal Cancer and Social Determinants of Health Screening [10]	26	completed	interventional	staff of community health centers	performance of colorectal cancer screening COR = 0.068 for the intervention analyzed, COR = 0.028 for the standard procedure
2.	behavioral	Multilevel Intervention Based on Colorectal Cancer (CRC) and Cervical Cancer Self-screening in Rural, Segregated Areas [11]	48	completed	interventional	women 50–65 years old	75% of patients who performed an immunochemical test using the kit they received vs. 1% of patients who underwent testing only upon recommendation
3.		Scaling CRC Screening Through Outreach, Referral, and Engagement (SCORE) [12]	4318	completed	interventional	persons 45–75 years old	29.9% vs. 9.6% of patients who participated in the diagnosis following the intervention in the 50–75 years group; 33.5% vs. 28.5% of patients who participated in the diagnosis following the intervention in the 45–49 years group;
4.		Mailed FIT Outreach 2022 [13]	5460	completed	interventional	adults 50–74 years old	21.2% and 20.3% of patients who participated in the screening after reminders were sent electronically or by letter vs. 14.6–18.0% of patients who participated in the screening despite not receiving reminders
5.		Effectiveness and Implementation of mPATH-CRC [14]	77,145	completed	interventional	primary healthcare professionals, patients aged 18 and over, patients 50–74 years old with indications of a colorectal cancer screening	0.9% of patients who completed a higher intensity program vs. 1.0% of patients who completed a lower intensity program
6.		Engaging Patients in Colon Cancer Screening Decisions During COVID-19 [15]	800	completed	interventional	adults 45–75 years old	35.1% of patients who underwent colonoscopy in the shared decision-making group vs. 22.8% in the control group.
7.		Helping Patients and Providers Make Better Decisions About Colorectal Cancer Screening [16]	1111	completed	interventional	adults 50–75 years old	35.1% of individuals who made a screening decision in the group of patients that received personalized screening messages vs. 31.0% of those who made a screening decision in the group of patients that only received information material
8.		Evaluating the Shared Decision Making Process Scale in Cancer Screening Decisions [17]	240	completed	observational	adults 35–80 years old	Variation in mean values on the decision participation scale between the colorectal diagnosis group and the prostate cancer diagnosis group with Cohen’s effect strength d = 0.90
9.		Test Up Now Education Programme [19]	115	completed	interventional	adults of African-American descent 45–64 years old	64.4% of patients in the group who met with the counsellor completed the study vs. 64.6% of patients in the control group
10.		e-Motivación: Developing and Pilot Testing an App to Improve Latinos’ Screening Colonoscopy Rates [20]	42	completed	interventional	adults of Latin-American descent	86.4% of patients who presented for colonoscopy in the intervention group vs. 95.0% in the control group
11.		Audio and Video Brochures for Increasing Colorectal Cancer Screening Among Adults Living in Appalachia [21]	94	completed	interventional	adults aged 50–64 residing in remote, hard-to-reach mountain towns	28.1% of those who took the immunochemical test supplied with video materials, 9.5% of those who took the test provided with audio materials, 15.0% of those who took the test without additional materials
12.		Virtual Human Delivered Nutrition Module for Colorectal Cancer Prevention [22]	139	completed	interventional	adults aged 45–73	Variation in mean values on the intention to take part in diagnostic tests scale between the group using the website and the group using the virtual assistant with a minimal effect strength—Cohen’s d = 0.13.
13.		Health Service Intervention for the Improvement of Access and Adherence to Colorectal Cancer Screening [23]	183	completed	interventional	adults aged 50–75	84.0% of patients rated the materials and assistance in performing an immunochemical test or colonoscopy as helpful
14.		The Prevent Anal Cancer Self-Swab Study [24]	253	completed	interventional	persons aged 25 or more, men who had anal sex with men	65.0% of men who received a home HPV test performed the test (vs. 52.5% of men who performed the HPV test in a medical facility); 63.4% of men who could perform the HPV test in a medical facility (vs. 51.2% of men who received the test at home) reported for an anoscopy
15.	Diagnosing	The Prevent Anal Cancer Palpation Study [25]	718	completed	interventional	persons aged 25 or more, men who had anal sex with men	concordance between the findings on palpation performed by the patients post-training and that performed by a medical professional of at least 95% and concordance between the findings on palpation performed by the patients’ partners post-training and that performed by a medical professional of at least 88.5%
16.		Patient Acceptance and Preference Among Screening Modalities for Detection of Barrett’s Esophagus [26]	24	completed	interventional	adults with gastroesophageal reflux disease or Barrett’s Esophagus	Variation in mean values on the pain analogue scale (VAS) between the group in which transnasal endoscopy was performed compared with the group in which cytosponge was utilized and the group in which traditional gastroscopy was performed with a very high Cohen’s d strength, d = 1.51 and d = 2.01, respectively. Percentage of patients willing to repeat the examination in the future: 87.5% in the cohort where transnasal endoscopy was performed and 100% in the other two groups
17.		Clinical Outcomes for Offering Genetic Testing in a Tiered Approach [27]	6	completed	interventional	oncologists	post-training 25/48 patients were correctly referred, pre-training 29/100 patients
18.		Endoscopic Optical Coherence Tomography for Screening and Diagnosis of Colorectal Precancerous and Malignant Polyps [28]	36	ongoing	interventional	adults aged 40 or more	the concordance between OCT-guided colonoscopy and biopsy results measured by Cohen’s k coefficient was high at =0.85.
19.		Colorectal Cancer and Pre-Cancerous Adenoma Non-Invasive Detection Test Study [29]	14,263	completed	interventional	adults aged 45 or more	The sensitivity of the test analyzed against colonoscopy results was 92.6%.

## 5. Limitations

The studies included in this review were conducted during the COVID-19 pandemic. Due to the public health situation, the number of studies carried out in this period may not align with those from other times. The shift in focus to other research priorities and concerns may have impacted the design and outcomes of the current research. Readers should consider that the timeline of the studies could have affected their progress and findings when reviewing the results. It is also crucial to note that while clinicaltrials.gov is one of the largest and most recognized databases, it does not capture all studies on gastrointestinal cancer screening and early detection. Since not all studies are registered in this database, our approach has the limitation of not including non-registered studies.

## 6. Conclusions

Most of the studies in this group of cancers were directed at colorectal cancer, which is the second greatest cause of cancer deaths worldwide. During the period analyzed, no studies were found on gastric cancer, although it has a high mortality rate, i.e., right after colorectal cancer.

Most of the studies analyzed dealt with the problem of test reporting. The results indicate that active patient involvement in the decision-making process increases commitment to screening. Also, the provision of self-administered stool immunochemical test kits for colorectal cancer screening proved to be considerably more effective in encouraging patient participation. Active methods of promoting screening programs such as assistance with enrolment and test reminders showed more effectiveness than educational materials alone. However, no advanced technology for providing interactivity was necessary.

As for new methods of early detection, research focused mainly on two topics: optical coherence tomography (OCT), used during colonoscopies, and an RNA test for stool examination in colorectal cancer. The results regarding both methods were promising.
